# 11-Oxygenated C19 steroids are the predominant androgens responsible for hyperandrogenemia in Cushing’s disease

**DOI:** 10.1530/EJE-22-0320

**Published:** 2022-09-29

**Authors:** Hanna F Nowotny, Leah Braun, Frederick Vogel, Martin Bidlingmaier, Martin Reincke, Lea Tschaidse, Matthias K Auer, Christian Lottspeich, Stefan A Wudy, Michaela F Hartmann, James Hawley, Joanne E Adaway, Brian Keevil, Katharina Schilbach, Nicole Reisch

**Affiliations:** 1 Medizinische Klinik and Poliklinik IV, Klinikum der Universität München, LMU München, Munich, Germany; 2 Division of Pediatric Endocrinology & Diabetology, Laboratory for Translational Hormone Analysis in Pediatric Endocrinology, Steroid Research & Mass Spectrometry Unit, Center of Child and Adolescent Medicine, Justus-Liebig-University, Giessen, Germany; 3 Department of Clinical Biochemistry, Manchester University Foundation NHS Trust, Manchester Academic Health Sciences Centre, Manchester, UK

## Abstract

**Background:**

Symptoms of hyperandrogenism are common in patients with Cushing’s disease (CD), yet they are not sufficiently explained by androgen concentrations. In this study, we analyzed the contribution of 11-oxygenated C19 steroids (11oxC19) to hyperandrogenemia in female patients with CD.

**Methods:**

We assessed saliva day profiles in females with CD pre (*n*  = 23) and post (*n*  = 13) successful transsphenoidal surgery, 26 female controls, 5 females with CD treated with metyrapone and 5 treated with osilodrostat for cortisol, cortisone, androstenedione (A4), 11-hydroxyandrostenedione (11OHA4), testosterone (TS), 11-ketotestosterone (11KT), as well as metabolites of classic and 11-oxygenated androgens in 24-h urine. In addition, morning baseline levels of gonadotropins and estradiol, sex hormone-binding globulin, cortisol and dehydroepiandrosterone sulfate (DHEAS) in serum and adrenocorticotrophic hormone in plasma in patients and controls were investigated.

**Results:**

Treatment-naïve females with CD showed a significantly elevated area under the curve of 11OHA4 and 11KT in saliva throughout the day compared to controls (11OHA4 mean rank difference (mrd) 18.13, *P* = 0.0002; 11KT mrd 17.42; *P* = 0.0005), whereas A4, TS and DHEAS were comparable to controls. Gonadotropin concentrations were normal in all patients with CD. After transsphenoidal surgery, 11oxC19 and their metabolites dropped significantly in saliva (11OHA4 *P* < 0.0001; 11KT *P* = 0.0010) and urine (11-oxo-androsterone *P* = 0.0011; 11-hydroxy-androsterone *P* < 0.0001), treatment with osilodrostat and metyrapone efficaciously blocked 11oxC19 synthesis.

**Conclusion:**

Hyperandrogenemia in CD is predominantly caused by excess of 11oxC19 steroids.

## Introduction

Cushing’s syndrome (CS) is characterized by typical signs and symptoms of excessive hypercortisolism with subsequent loss of normal feedback mechanisms of the hypothalamic–pituitary–adrenal (HPA) axis and cortisol circadian rhythmicity (1). In up to 80% of cases, it is the result of excessive adrenocorticotrophic hormone (ACTH) secretion mostly by pituitary adenomas, referred to as Cushing’s disease (CD) (2, 3, 4).

First-line therapy in CD is surgical resection of the adenoma, mostly transsphenoidal adenomectomy. However, remission is only achieved in about 71–80% (5, 6). Medical therapy is an option if surgery is not successful and includes pituitary-directed drugs, glucocorticoid receptor-directed drugs and adrenal-directed drugs, such as metyrapone and osilodrostat, which both inhibit steroidogenesis (5, 7).

In our study, the focus is set on hyperandrogenemia in CD. Menstrual irregularities, acne, hirsutism and telogen effluvium are common symptoms in patients with CD often resembling the clinical phenotype of polycystic ovary syndrome (PCOS). Hyperandrogenism in CD is owing to multiple causes. One main reason is that hyperandrogenemia is thought to be the result of a direct elevation of adrenal androgens due to ACTH stimulation (8, 9, 10, 11, 12). The dominant role of ACTH in the regulation of adrenal androgens readily explains the differences in concentrations of androgen precursors as androstenedione (A4), dehydroepiandrostendione (DHEA) (13) and dehydroepiandrosterone-sulphate (DHEAS) in different subtypes of CS with the highest concentrations of adrenal androgens in CS due to ectopic ACTH production and low-to-suppressed adrenal androgens in CS due to an adrenal adenoma (10, 14). However, the symptoms of hyperandrogenism in CD are not sufficiently explained by the measured androgen concentrations. Instead, there seems to be a clear dissociation of cortisol and adrenal androgen secretion in CD. In most patients with CD, DHEAS concentrations have been found to be in the high normal range but only rarely elevated (12). In addition, no difference in adrenal androgen concentrations has been observed in hirsute and non-hirsute females with CD (12). Lado-Abeal *et al.* found significant and inverse correlations of serum estradiol (E2) and serum cortisol levels, but no correlation was observed with serum androgens with menstrual irregularities in females with CD (15). They concluded that menstrual irregularities in CD are the result of hypercortisolemic inhibition of gonadotropin release acting at the hypothalamic level, rather than raised androgen concentrations (15), although they did not find altered gonadotropin concentrations.

We hypothesized that the main drivers of the hyperandrogenic phenotype in patients with CD might be elevated concentrations of 11-oxygenated C19 adrenal-derived steroids (11oxC19). The synthesis of these 11oxC19 steroids requires 11β-hydroxylase (CYP11B1) which means that they are adrenal-specific and stimulated by ACTH (16). 11-Ketotestosterone (11KT) and 11-ketodihydrotestosterone (11KDHT) have been shown to be active androgens with equal potency at the androgen receptor as testosterone (TS) and dihydrotestosterone (DHT), respectively (17). As osilodrostat and metyrapone inhibit CYP11B1, we also analyzed the effect of osilodrostat and metyrapone treatment on the concentrations of 11oxC19 steroids in patients with CD.

This is the first study to investigate the significance of 11-oxygenated androgens in CD pre and post transspenoidal surgery and under metyrapone and osilodrostat.

## Subjects and methods

### Subjects

Patients were recruited from the Endocrine Outpatient Clinic of the University Hospital Munich, Germany. All patients had clinically and biochemically confirmed CD and provided written informed consent to participate in the German Cushing’s Registry (NeoExNET, ethical approval no. 152-10, ethics committee of the medical faculty of the Ludwig Maximilians University Munich). The control group was selected from the control group of the German Cushing’s Registry (overweight to obese females (median BMI 26.7 kg/m^2^ (IQR: 8.8), medium age of 31.5 years (IQR: 17.0), who underwent extensive exclusion of hypercortisolism by 24-h urine, salivary cortisol and dexamethasone suppression test (DST)).

This study included 59 females of which 23 were patients with treatment-naïve CD, 5 patients with CD and treatment with osilodrostat and 5 patients on metyrapone (1 patient received metyrapone first and later osilodrostat; 1 patient received a combination of metyrapone and ketokonazole) and 26 healthy female controls.

Saliva samples (Salivettes; Sarstedt, Nümbrecht, DE, Germany) at five timepoints (Tn) throughout the day, namely 08:00, 12:00, 16:00, 20:00 and 22:00 h) and 24-h urine were collected according to a standardized protocol.

Incomplete data sets in saliva samples made up 39.0% and were mainly attributed to sporadic analytical interference in data analysis or insufficient sample volume in single samples of the day profile. For values lower than the lower limit of quantitation (LLOQ), values were equated to the respective LLOQ.

### Steroid hormone analysis

A validated liquid chromatography–mass spectrometry (LC–MS/MS) assay was used for the simultaneous measurement of cortisol, cortisone, TS, A4, 11β-hydroxyandrostenedione (11OHA4) and 11KT in saliva as previously described (14, 18, 19).

Gas chromatography-mass spectrometry (GC-MS) analysis was used to quantify concentration of steroid metabolites (TS; An, androsterone; 11-O-An, 11-oxo-androsterone and 11-OH-AN, 11-hydroxy-androsterone) in 24-h urinary specimens as previously described (20, 21, 22).

Blood concentrations of luteinizing hormone (LH), follicle-stimulating hormone (FSH), E2, sex hormone-binding globulin (SHBG), DHEAS, cortisol, ACTH and TS were analyzed using the following assays and reference ranges: SHBG 21–138 nmol/L; DHEAS 0.2–4 µg/mL; cortisol 1.8–24 µg/dL; ACTH 4–61 pg/mL; total TS 14–69 ng/dL. TS and SHBG: CLIA, IDS-iSYS, Immunodiagnostic Systems, Boldon, UK; DHEAS, cortisol and ACTH: CLIA, Liaison, DiaSorin, Saluggia Italy.

### Statistical analysis

Data were tested for normality using the Shapiro–Wilk test. Column statistics were calculated using GraphPad Prism (median, interquartile range (IQR), mean, s.e.m., quartiles). Normally distributed data were analyzed using unpaired *TS*-test or one-way ANOVA. Non-normally distributed data were analyzed using Mann–Whitney test or Kruskal–Wallis test and Dunn’s multiple comparisons test. Area under the curve (AUC) was calculated with baseline Y = 0, positive peak direction and ignoring peaks that are less than 10% of the distance from minimum to maximum Y using GraphPad Prism. For correlation analysis, Spearman’s correlation coefficient was computed for non-parametric data. The CI was defined as 95% and a *P*-value of <0.05 was considered statistically significant *P* ≤ 0.05 (≤ 0.05 (*), ≤ 0.01 (**), ≤ 0.001 (***), < 0.0001 (****)). Statistical analysis and graphical presentation were carried out using GraphPad Prism 7.03 and Adobe Illustrator 2020.

## Results

### Characteristics of study participants

Patients presented with CD (confirmed by urinary free cortisol (UFC) (median: 665 µg/24 h (IQR: 516) and DSTwith median cortisol of 12.4 µg/dL (IQR: 11.8)) due to pituitary adenoma, in most cases microadenoma. All patients underwent transsphenoidal surgery as a first treatment option, with a successful outcome in 13/23 cases. Patients with persistence of the disease were successfully treated with metyrapone (median dose of 1625 mg/day (IQR: 1125), median UFC: 42.2 µg/24 h (IQR: 125.4)) or osilodrostat (4 mg/day (IQR: 8.5), median UFC 60.8 µg/24 h (IQR: 152.4)).

As depicted in Table 1, the median age was 43.0 years (IQR: 18.0) in CD patients treated with osilodrostat, 47.0 years (IQR: 16.0) in patients treated with metyrapone, 37.0 years (IQR: 13.0 years) in treatment-naïve CD patients and 31.5 years (IQR: 17.0) in female controls. The age of CD patients treated with metyrapone was higher than that in healthy controls (Dunn’s multiple comparisons test mean rank difference = 23.96, *P* = 0.0255).

**Table 1:** Patient characteristics. Values are presented as median (IQR).

	Osilodrostat	Metyrapone	Treatment naïve	Controls	*P*-value
*n*	5	5	23	26	
Age, years	43.0 (18.0)	47.0 (16.0)	37.0 (13.0)	31.5 (17.0)	0.0210
BMI (kg/m^2^)	29.7 (5.3)	34.8 (12.5)	29.3 (10.2)	26.7 (8.8)	0.3517
UFC (µg/24 h)	60.8 (152.4)	42.2 (125.4)	665 (516)	WRR	0.0001
DST (µg/dL)			12.4 (11.8)	WRR	

DST, dexamethasone suppression test; UFC, urinary free cortisol; WRR, within reference range.

Median BMI was comparable between all groups (*P*  = 0.3517): 29.7 kg/m^2^ (IQR: 5.3) in osilodrostat treated patients, 34.8 kg/m^2^ (IQR: 12.5) in metyrapone-treated patients, 29.3 kg/m^2^ (IQR: 10.2), in treatment-naïve CD patients and 26.7 kg/m^2^ (IQR: 8.8) in controls.

Median morning ACTH of treatment-naïve CD patients was 51.0 pg/mL (IQR: 50.0) vs 10.0 pg/mL (IQR: 7.8) in controls, *P* < 0.0001); median morning cortisol in serum was 20.7 nmol/L (IQR: 10.2) vs 10.2 nmol/L (IQR: 9.3) in healthy controls, *P* < 0.0001.

### 11oxC19 steroids are the main androgens in patients with CD

**Figure 1:**
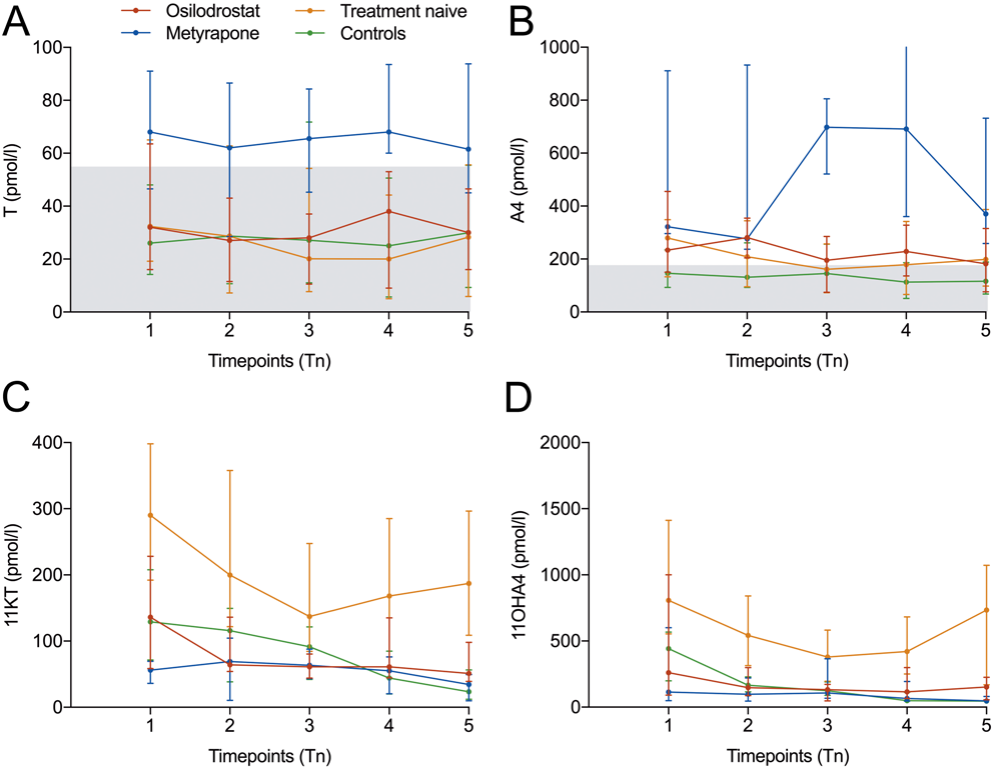
Levels of 11oxC19 steroids 11KT and 11OHA4 are elevated in treatment-naïve CD patients compared to controls, while classical androgens are elevated under metyrapone treatment. (A) TS, (B) A4, (C) 11KT and (D) 11OHA4 measurements (pmol/L) of CD patients without treatment (*n*  = 23), on treatment with osilodrostat (*n*  = 5) or with metyrapone (*n*  = 5) and those of healthy female controls (*n*  = 26) are plotted at five different timepoints throughout the day (Tn 1–5). Data are presented as median and IQR. The reference interval for TS and A4 is shaded in gray .

A clear elevation of 11KT and 11OHA4 concentrations over the whole day was observed in treatment-naïve patients (compare Fig. 1C, D and Table 2) compared to controls with an unusual nighttime increase in 11oxC19 concentrations, while median A4 and TS levels were not significantly different (compare Fig. 1A, B and Table 2).

**Table 2:** Comparison of TS, A4, 11KT and 11OHA4 concentrations at timepoints 1–5 in treatment-naïve CD patients vs controls. Median (IQR) of treatment-naïve CD patients (Tn) vs controls (C) in pmol/L.

Timepoint	TS	*P*	A4	*P*	11KT	*P*	11OHA4	*P*
1		0.3998		0.0553		0.0002		0.0002
Tn	32.4 (45.8)		280.0 (216.0)		290.2 (206.0)		807.4 (874.1)	
C	26.0 (33.9)		146.4 (141.2)		129.2 (136.4)		442.1 (370.9)	
2		0.8969		0.2995		0.0004		0.0001
Tn	28.6 (55.6)		208.9 (249.2)		200.0 (235.8)		542.3 (537.9)	
C	28.7 (51.7)		130.9 (168.0)		115.9 (111.1)		165.4 (244.5)	
3		0.4519		0.7434		0.0036		0.0001
Tn	20.2 (46.6)		160.9 (184.4)		137.2 (163.0)		378.4 (426.5)	
C	27.1 (60.8)		145.4 (182.4)		91.34 (79.4)		123.5 (119.2)	
4		0.8229		0.2082		0.0001		0.0001
Tn	20.0 (39.2)		179.1 (275.9)		168.3 (209.0)		421.0 (550.0)	
C	25.0 (44.9)		113.1 (135.3)		44.2 (64.9)		49.6 (69.4)	
5		0.8907		0.0601		0.0001		0.0001
Tn	28.3 (49.8)		198.9 (289.1)		187.2 (187.8)		734.0 (912.6)	
C	30.0 (46.1)		116.3 (121.1)		23.6 (44.3)		45.0 (33.9)	

**Figure 2:**
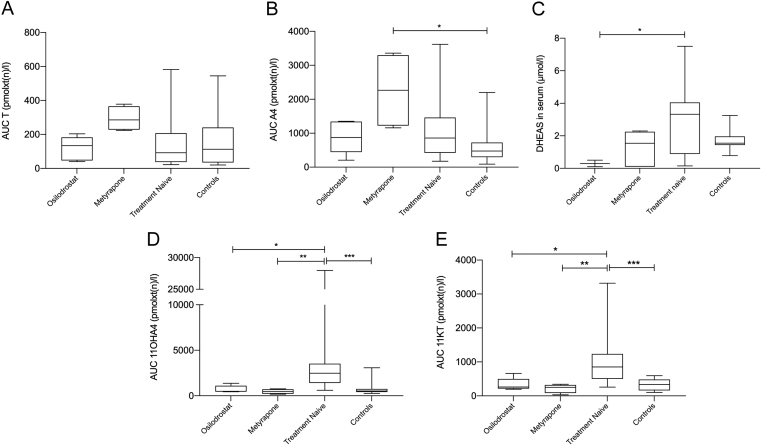
Levels of 11oxC19 AUCs are elevated in treatment-naïve CD patients compared to controls, while AUC of A4 is elevated under metyrapone treatment. AUCs of all CD patients without treatment (*n*  = 23), on treatment with osilodrostat (*n*  = 5) or with metyrapone (*n*  = 5) and those of healthy female controls (*n*  = 26) were calculated. Data sets with missing TP1 or TP5 were excluded from AUC calculation. The five box plots represent the AUCs of (A) TS, (B) A4, (C) DHEAS, (D) 11KT and (E) 11OHA4. Kruskal–Wallis test and Dunn’s multiple comparisons test were used for statistical analysis. *P*-value ≤ 0.05 (*), ≤ 0.01 (**), ≤ 0.001 (***).

This is emphasized by a significant increase of the respective AUCs of 11KT and 11OHA4 in treatment-naïve patients with CD compared to healthy controls (Fig. 2D, E and Table 3; median 11KT-AUC in treatment-naïve CD 853.2 pmolxt(n)/L (IQR: 731.8) vs 334.7 pmolxt(n)/L (IQR: 321.7) in controls, *P* < 0.0001; median 11OHA4-AUC 2484.0 pmolxt(n)/L (IQR: 2122.0) vs 530.4 pmolxt(n)/L (IQR: 336.0), *P* < 0.0001). However, there was no significant difference between AUCs of classical androgens TS or A4 and controls (median AUC of TS in treatment-naïve CD patients 92.2 pmolxt(n)/L (IQR: 170.0) vs 112.7 pmolxt(n)/L (IQR: 205.5) in controls, *P* = 0.9813; median AUC of A4 in treatment-naïve CD patients 858.2 pmolxt(n)/L (IQR: 1040.7) vs 476.6 pmolxt(n)/L (IQR: 431.8) in controls, *P* = 0.0796; compare Fig. 2A, B and Table 3). DHEAS levels in serum were not significantly different in treatment-naïve CD patients vs controls (median in treatment-naïve CD patients 3.3 µmol/L (IQR: 3.2) vs 1.6 µmol/L (IQR: 0.5) in controls, *P* = 0.0708; compare Fig. 2C).

**Table 3:** Comparison of AUC of TS, A4, 11KT and 11OHA4 in treatment-naïve CD patients (Tn) vs controls (C). Values are median AUC (IQR).

	AUC (pmolxt(n)/L)		*P*-value
	Tn	C	
TS	92.2 (170.0)	112.7 (205.5)	0.9813
A4	858.2 (1040.7)	476.6 (431.8)	0.0796
11KT	853.2 (731.8)	334.7 (321.7)	0.0001
11OHA4	2484.0 (2122.0)	530.4 (336.0)	0.0001

Of the 23 patients with treatment-naïve CD, 6 patients presented with no hyperandrogenic symptoms, 6 with 1 symptom, 6 with 2 symptoms, 4 patients with 3 symptoms and only 1 patient with all 4 symptoms of clinical hyperandrogenism. A positive trend, but no significant positive correlation between the symptoms of hyperandrogenism and 11oxC19 steroids was detected.

### Correlation of cortisol/cortisone concentrations with ACTH, E2 and gonadotropin concentrations and androgens in CD patients

**Figure 3:**
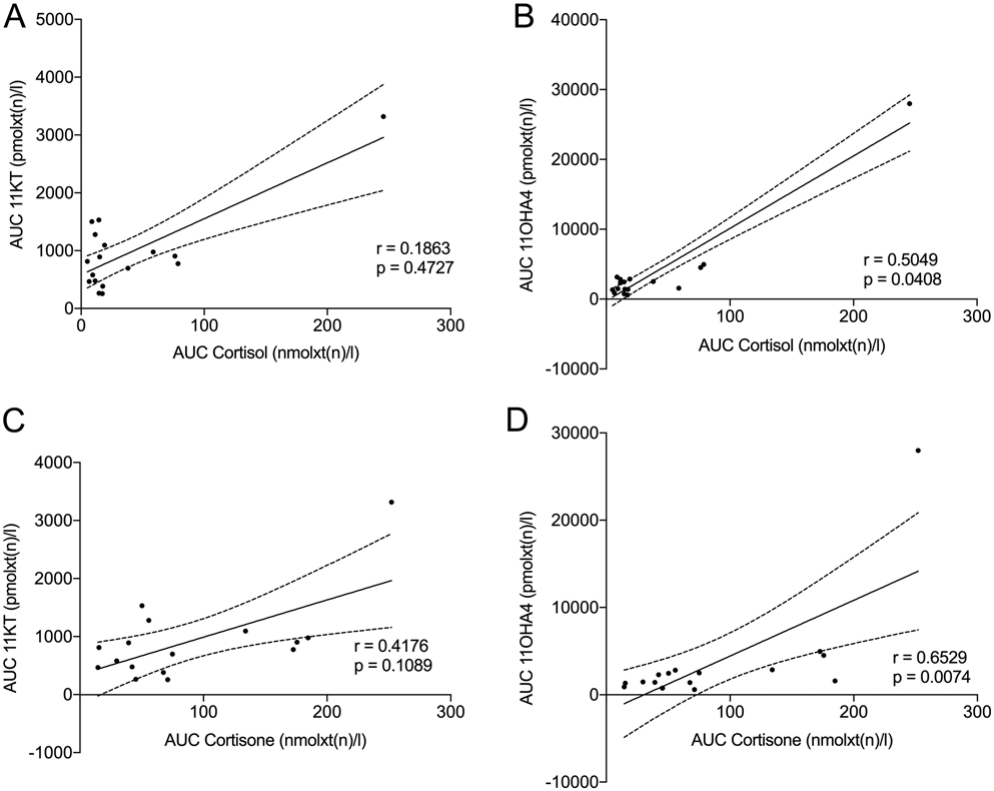
AUC of 11oxC19 steroids correlate with cortisol/cortisone levels in treatment-naïve CD patients. AUCs of (A and C) 11KT and (B and D) 11OHA4 of all CD patients without treatment (*n*  = 23) were calculated. Data sets with missing TP1 or TP5 were excluded from the AUC calculation. Correlation of 11oxC19 steroids with (A and B) cortisol and (C and D) cortisone was calculated. For non-parametric data analysis, Spearman’s correlation coefficient was computed. The black line indicates the line of best fit (unknowns are interpolated from standard curve) and the dotted lines represent the 95% CI. *P* ≤ 0.05 (≤ 0.05 (*), ≤ 0.01 (**), ≤ 0.001 (***), < 0.0001 (****)).

AUC of salivary cortisol correlated significantly with 11OHA4 (r = 0.5049, *P* = 0.0408), A4 (r = 5941, *P* = 0.0172) and ACTH (r = 0.4640, *P* = 0.0454), while there was no significant correlation to 11KT (r = 0.1863, *P* = 0.4727), TS ( r = 0.4294, *P* = 0986), gonadotropins (LH r = −0.2239, *P* = 0.4016; FSH r = 0.0339, *P* = 0.9020), E2 (r = −0.2107, *P* = 0.4499), SHBG (r = −0.5324, *P* = 0.0775) and DHEAS (r = 0.5364, *P* = 0.0939) as demonstrated in Fig. 3 and Table 4. Serum cortisol levels showed a significant correlation with 11OHA4 (r = 0.5000, *P* = 0.0293; data not shown). As salivary cortisone was demonstrated to be a more accurate estimate for serum cortisol concentrations, we also included a correlation analysis of all hormones with AUC of salivary cortisone (23). We confirmed the positive correlation of AUC of salivary cortisone with ACTH (r = 0.7344, *P* = 0.0017), A4 (r = 0.6893, *P* = 0.0057) and 11OHA4 concentrations (r = 0.6529, *P* = 0.0074) as well as a positive correlation with DHEAS concentrations in serum (r = 0.7167, *P* = 0.0369) as shown in Fig. 3 and Table 4.

**Table 4:** Correlation of cortisol and cortisone with other hormone parameters in treatment-naïve CD patients. Statistically significant values are presented in bold.

	Cortisol		Cortisone	
	*r*^†^	*P*	*r*^†^	*P*
LH (U/L)	−0.2239	0.4016	−0.2503	0.4066
FSH (U/L)	0.0339	0.9020	0.0220	0.9493
E2 (pg/mL)	−0.2107	0.4499	−0.2168	0.4990
SHBG (nmol/L)	−0.5324	0.0775	−0.0500	0.9116
ACTH (pg/mL)	**0.4640**	**0.0454**	**0.7344**	**0.0017**
DHEAS (µmol/L)	0.5364	0.0939	**0.7167**	**0.0369**
Saliva AUC, pmolxt(n)/L				
TS	0.4294	0.0986	0.4786	0.0735
A4	**0.5941**	**0.0172**	**0.6893**	**0.0057**
11KT	0.1863	0.4727	0.4176	0.1089
11OHA4	**0.5049**	**0.0408**	**0.6529**	**0.0074**

^†^Spearman’s correlation coefficient.

### Transsphenoidal surgery of patients with Cushing’s disease due to pituitary adenoma efficaciously decreases 11oxC19 concentrations

**Figure 4:**
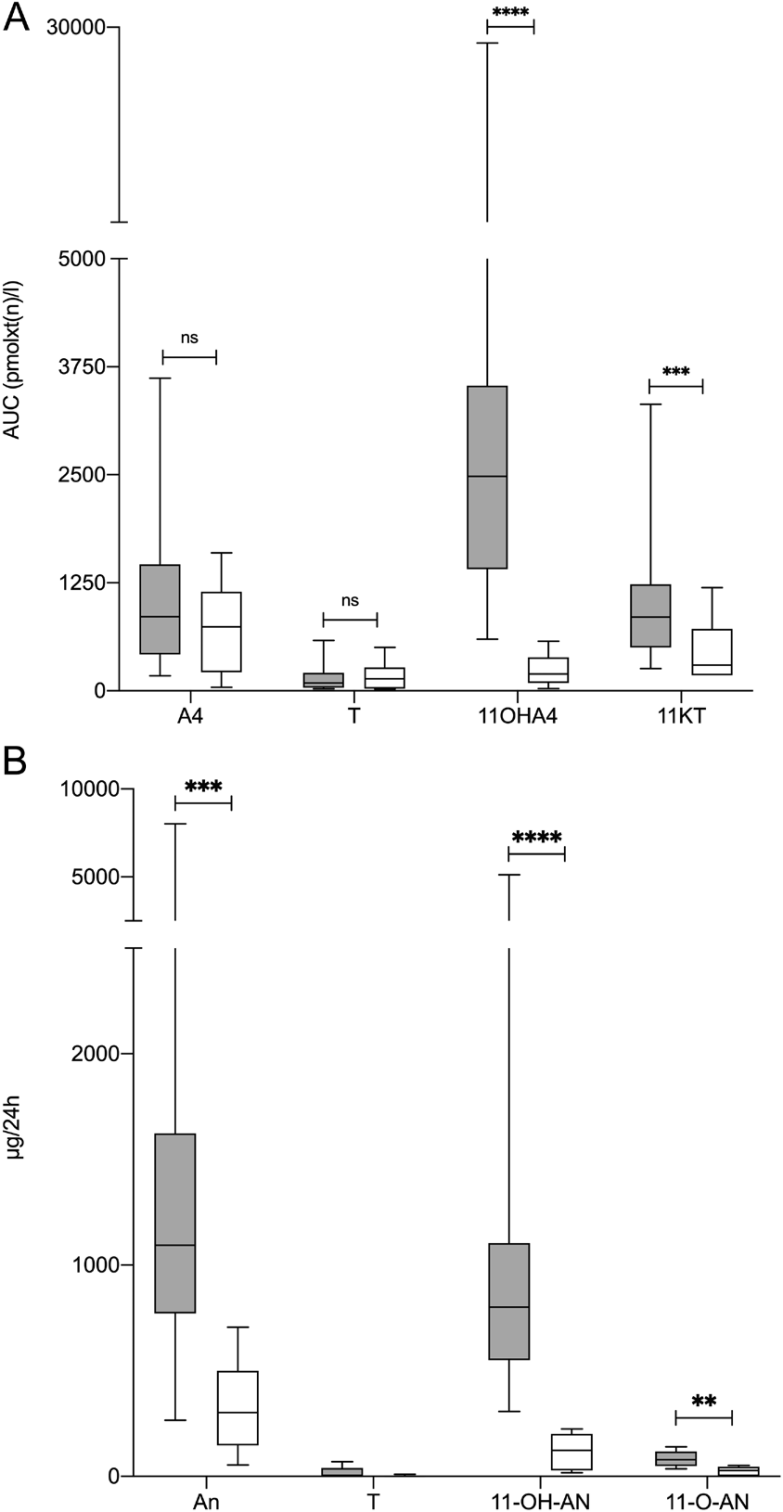
11oxC19 levels are significantly reduced after transsphenoidal surgery. (A) Salivary AUCs of TS, A4, 11OHA4 and 11KT of all CD patients without treatment ( gray boxes, *n*  = 23) were calculated and compared to AUCs of TS, A4, 11OHA4 and 11KT after successful transsphenoidal surgery (white boxes, *n*  = 13/23). Mann–Whitney test was used for statistical analysis of pre- vs postoperative measurements. (B) 24-h steroid metabolite analysis was performed in 14 CD patients before ( gray boxes) and 7 patients after (white boxes) transsphenoidal surgery. Urinary metabolites of classical androgens included An, androsterone and TS, testosterone. Urinary metabolites of 11oxC19 androgens included measurement of 11-OH-AN, 11-hydroxy-androsterone and 11-O-AN, 11-oxo-androsterone. Wilcoxon matched-pairs signed-rank test was used for statistical analysis of pre- vs postoperative measurements. Data are presented as a boxplot diagram including median and IQR. *P* ≤ 0.05 (≤ 0.05 (*), ≤ 0.01 (**), ≤ 0.001 (***), < 0.0001 (****)).

Salivary profiles after transsphenoidal surgery revealed an efficacious reduction of 11oxC19 concentrations compared to pre-operative steroid AUC (compare Fig. 4A; 11OHA4 pre-operative 2484.00 pmolxt(n)/L (IQR: 2122.00) vs post-operative 196.00 pmolxt(n)/L (IQR: 293.80) with *P* < 0.0001 and 11KT pre-operative 853.20 pmolxt(n)/L (IQR: 731.80) vs post-operative 297.00 pmolxt(n)/L (IQR: 536.80) with *P* = 0.0010), but there was no significant difference between AUC of classical androgens (TS pre-operative 92.18 pmolxt(n)/L (IQR: 196.96) vs post-operative 137.50 pmolxt(n)/L (IQR: 242.75) with *P* = 0.6690 and A4 pre-operative 858.20 pmolxt(n)/L (IQR: 1040.70) vs post-operative 739.50 pmolxt(n)/L (IQR: 861.00) with *P* = 0.3719). This marked elevation of 11oxC19 steroids pre-surgery and significant reduction post-surgery correlated with the post-surgical reduction in ACTH (delta AUC 11OHA4 vs delta ACTH, *P* = 0.0097; delta AUC 11KT vs delta ACTH, *P* = 0.0475) and was confirmed in 24-h urinary steroid metabolite analysis (compare Fig. 4B; 11-OH-AN pre-operative 800.4 µg/24 h (IQR: 554.1) vs post-operative 122.4 µg/24 h (IQR: 173.77) with *P* < 0.0001 and 11-O-AN pre-operative 79.0 µg/24 h (67.7) vs post-operative 27.68 µg/24 h (IQR: 45.43) with *P* = 0.0011).

### Osilodrostat treatment reduces androgen concentrations more efficaciously compared to metyrapone

In addition, we investigated the effect of steroidogenesis inhibitors osilodrostat and metyrapone on concentrations of classical and 11oxC19 steroids.

Concentrations of 11oxC19 steroids were normalized with equal efficacy by osilodrostat and metyrapone (Fig. 2D and E, median 11KT in treatment-naïve CD patients (853.2 pmol/L, IQR 731.8) vs metyrapone (251.0 pmol/L (IQR: 237.4) *P* = 0.0096) and vs osilodrostat group (261.5 pmol/L (IQR: 282.8) *P* = 0.0375); median 11OHA4 in treatment-naïve CD patients (2484.0 pmol/L, IQR: 2122.0) vs metyrapone (461.5 pmol/L, IQR: 500.9, *P* = 0.0072) and vs osilodrostat group (492.0 pmol/L, IQR: 642.7, *P* = 0.0304)).

In patients on metyrapone, but not on osilodrostat, classical androgens – especially A4 – were markedly elevated throughout the whole day compared to healthy controls and treatment-naïve CD patients (Figs 1A, B, 2B and Table 5, median A4 on metyrapone (2266 pmol/L (IQR: 2072)) vs healthy controls (476.6 pmol/L (IQR: 431.8), *P* = 0.0013 and vs treatment-naïve CD 858.2 pmol/L (IQR: 1040.7) *P* = 0.0365). Interestingly, median concentration of DHEAS on osilodrostat was reduced (0.3 µmol/L (IQR: 0.4)) vs treatment-naïve CD patients 3.3 µmol/L (IQR: 3.2), *P* = 0.0349; Fig. 2C.

**Table 5:** Comparison of TS and A4 concentrations at timepoints 1–5 in metyrapone- or osilodrostat-treated patients vs treatment-naïve CD patients or and vs controls. Values are presented as median (IQR).

Timepoint	TS (pmol/L)				A4 (pmol/L)			
	Metyrapone		Osilodrostat		Metyrapone		Osilodrostat	
	Values	*P*	Values	*P*	Values	*P*	Values	*P*
1								
TP	68.0 (44.5)		32.0 (47.5)		322.0 (614.5)		234.0 (305.0)	
Tn	32.4 (45.8)	0.5338	32.4 (45.8)	>0.9999	280.0 (216.0)	0.4389	280.0 (216.0)	>0.9999
C	30.7 (44.0)	0.4331	30.7 (44.0)	>0.9999	166.9 (137.8)	0.0391	166.9 (137.8)	>0.9999
2								
TP	62.0 (58.0)		27.0 (31.5)		276.0 (696.0)		281.5 (150.3)	
Tn	28.6 (55.6)	0.8627	28.6 (55.6)	>0.9999	208.9 (249.2)	0.4147	208.9 (249.2)	>0.9999
C	33.9 (51.7)	>0.9999	33.9 (51.7)	>0.9999	155.7 (167.7)	0.1199	155.7 (167.7)	0.9158
3								
TP	65.5 (39.0)		28.0 (26.5)		697.5 (284.8)		195.0 (211.0)	
Tn	20.2 (46.6)	0.2696	20.2 (46.6)	>0.9999	160.9 (184.4)	0.0148	160.9 (184.4)	>0.9999
C	27.1 (58.4)	0.8532	27.1 (58.4)	>0.9999	154.1 (194.9)	0.0109	154.1 (194.9)	>0.9999
4								
TP	68.0 (33.5)		38.0 (44.0)		691.0 (751.0)		229.0 (192.0)	
Tn	20.0 (39.2)	0.0382	20.0 (39.2)	>0.9999	179.1 (275.9)	0.0402	179.1 (275.9)	>0.9999
C	27.6 (55.4)	0.1512	27.6 (55.4)	>0.9999	116.9 (211.3)	0.0056	116.9 (211.3)	>0.9999
5								
TP	61.5 (48.8)		30.0 (30.5)		370.0 (473.3)		182.0 (239.0)	
Tn	28.3 (49.8)	0.3576	28.3 (49.8)	>0.9999	198.9 (289.1)	0.6719	198.9 (289.1)	>0.9999
C	32.7 (52.2)	0.5995	32.7 (52.2)	>0.9999	126.2 (112.6)	0.0866	126.2 (112.6)	>0.9999

C, controls; Tn, treatment-naïve CD patients; TP, metyrapone- or osilodrostat-treated patients.

There was no correlation between cortisol AUC and doses of osilodrostat (r = −0.6669, *P* = 0.2667) or metyrapone (r = 0.7379, *P* = 0.3333).

## Discussion

Previous studies show a dissociation of clinical symptoms of hyperandrogenism and measured androgen concentrations in CD (12, 15). Here we show that the degree of hyperandrogenemia in CD simply remained undetected as it is not mainly caused by classical androgens but by 11oxC19 steroids that are not (yet) routinely measured.

Our data clearly support the idea that 11oxC19 steroids are the major source of androgens in patients with CD responsible for hyperandrogenemia in CD.

Our data show that concentrations of 11oxC19 steroids 11KT and 11OHA4 are increased in female patients with treatment-naïve CD, while classical androgen precursors and androgens as A4, DHEAS and TS were not different from controls (compare Figs 1 and 2). 11OHA4 has been shown to be the second most abundant unconjugated adrenal androgen after DHEA (16). Steroid 11OHA4 is then further metabolized to 11KT and 11KDHT, which show equal androgenetic capacity as their counterparts T and DHT (17). Rege *et al.* have demonstrated a significant increase in DHEA, A4 and the 11oxC19 steroid 11OHA4 in the adrenal vein after ACTH administration in subjects with presumably aldosterone producing adrenal tumors. Also, active androgens such as TS and the 11oxC19 steroids 11β-hydroxytestosterone or 11KT were measured after ACTH stimulation, but to a lesser degree (24). This can be explained by the fact that 11KT and 11KDHT are primarily derived from peripheral conversion, and thus are detected to a lesser degree in adrenal vein sampling (25). Synthesis of 11OHA4, however, mainly takes place in the zona reticularis of the adrenal cortex (16). We conclude that the direct stimulation of 11oxC19 androgen synthesis by ACTH in CD is a relevant factor of hyperandrogenemia in this patient cohort. This is supported by the fact of a clear correlation of cortisol and cortisone with 11OHA4 concentrations and also cortisol/cortisone and ACTH concentrations in our study (Table 4). Furthermore, we show that the 11oxC19 steroids in saliva as well as their urinary metabolites significantly decreased after successful transsphenoidal surgery correlating with the decrease in ACTH, whereas there was no correlation of ACTH and classical androgens.

Menstrual irregularities in CD previously were also explained by direct inhibition of hypercortisolism on gonadotropin release acting at the hypothalamic level (15, 26). As menstrual irregularities were found to correlate with cortisol concentrations at two time points during the day, but not with androgen concentrations, the authors concluded that interference of cortisol with the HPG axis rather than raised circulating androgens cause menstrual disturbances in patients with CD even though cortisol did not correlate with gonadotropins nor did patients with irregular cycles show altered gonadotropin concentrations (15). The clear correlation of 11-oxygenated androgens with cortisol and cortisone in our study explains this phenomenon.

We did not discover any correlation between levels of LH, FSH and other measured hormones. We therefore conclude that disturbances of the HPA axis via the negative feedback loop of elevated cortisol or aromatized estrogen concentrations do not play a key factor in the etiology of menstrual disturbances in CD. Interestingly, the 11oxC19 steroids, however, have only been shown to be aromatizable *in vitro* and *ex vivo*. 11-oxygenated estrogens were not detectable in women with elevated 11oxC19 steroids or high aromatase concentrations (27). Hyperandrogenic phenotype therefore might rather be explained by direct androgenic effects of 11oxC19 steroids on the affected organs such as skin, hair or ovary instead of effects of aromatized estrogens on the HPG axis.

The lack of correlation of 11-oxygenated androgens and clinical hyperandrogenism in our study most likely is due to low sample size and insufficient and inaccurate documentation of hyperandrogenic symptoms due to cosmetic measures (data not shown). On the one hand, there can also be further causes of acne, hirsutism, telogen effluvium and menstrual disturbances than just a pure rise in androgen levels. One further limitation might be the fact that, even when 11oxC19 steroids act at the androgen receptor with equal potency as classical androgens (17), the downstream signaling cascade might be different for 11oxC19 and classical androgens.

**Figure 5:**
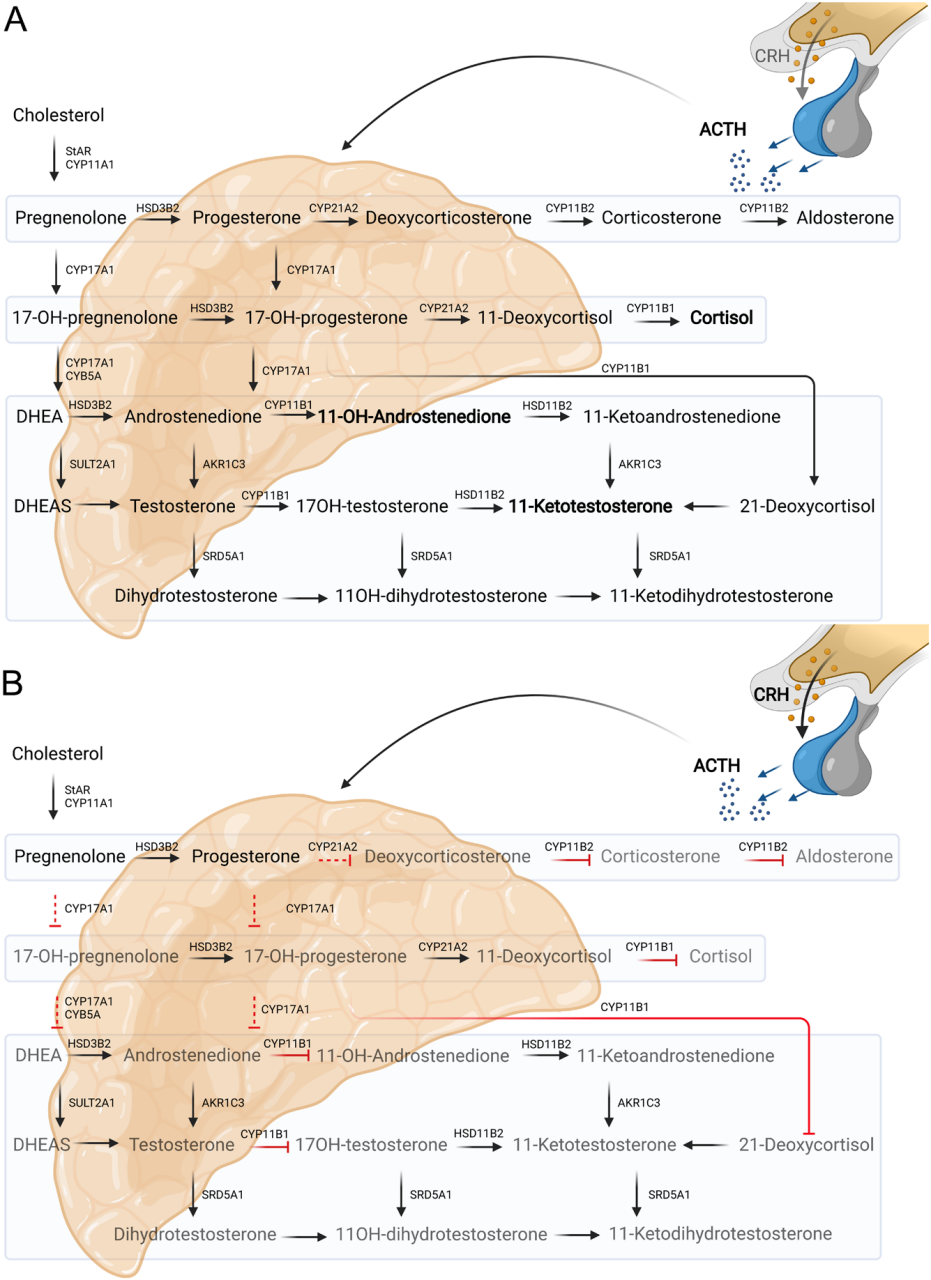
Adrenal steroidogenesis and the effect of the two steroidogenesis inhibitors metyrapone and osilodrostat. (A) Pathway of adrenal steroidogenesis including 11oxC19 androgen synthesis. (B) Effect of the steroidogenesis inhibitors metyrapone and osilodrostat on adrenal steroidogenesis including inhibition of CYP11B1, CYP11B2 and also the cholesterol side chain cleavage complex, 17α-hydroxylase and 21-hydroxylase.

Besides normalization of 11oxC19 by transspenoidal surgery, we show that this can also be achieved by treatment with osilodrostat and metyrapone (compare Figs 1C, D and 2D, E) as they both efficaciously block CYP11B1 and thus 11oxC19 synthesis (compare Fig. 5A and B).

Steroid CYP11B1 inhibition, however, also leads to an accumulation of 11-deoxycortisol and the classical androgen precursors DHEA and A4 and subsequent accelerated classical androgen synthesis (28, 29, 30).

Interestingly, metyrapone and osilodrostat seem to have a differential effect on the synthesis of classical androgens in patients with CD. Creemers *et al.* previously showed that A4 and TS accumulated more strongly in metyrapone-treated human adrenocortical cells compared to those treated with osilodrostat. Under osilodrostat treatment, even a slight decrease in levels of adrenal androgens was observed (31). This might be due to a stronger 17α‑hydroxylase (CYP17A1) and potentially cholesterol side chain cleavage enzyme and/or StaR protein inhibition, which has been proposed for osilodrostat (31) and verified in a recent publication. Bonnet-Serrano *et al.* showed that in patients on osilodrostat but not on metyrapone, 21-hydroxylase and CYP17A1 activity were significantly decreased (32). In our small subgroup of patients on metyrapone, but not on osilodrostat medication, treatment also led to a significant elevation of classical androgens, in particular of A4, thus, confirming the *in vitro and in vivo* results. In line, increased concentrations of TS were only observed in 4 of the 14 female patients treated with osilodrostat in the phase II study (28) and were described to have increased from a mean of 1.3 to 2.6 nmol/L in the phase III study (33). Differences of drug sensitivity on tissue levels may further modulate differences between various inhibitors of steroidogenesis as well as interindividual differences in clinical response to the same inhibitor (31).

The shortcomings of our study are the retrospective character and the small number of patients under treatment with steroidogenesis inhibitors due to the rarity of the disease. The strengths of the study are circadian steroid measurements via saliva day profiling pre- and post-surgery at home preventing in-hospital bias confirmed by metabolites in 24-h urine. Previously a strong correlation of serum and saliva hormone concentrations with regard to the measured hormones has been shown (18, 19).

To conclude, our data show that ACTH-mediated 11oxC19 steroids represent the predominant active androgens causing hyperandrogenemia in CD (Fig. 5). Our data also indicate that osilodrostat might be a more suitable steroidogenesis inhibitor for CD patients with clinical signs of hyperandrogenism. Prospective studies in larger cohorts are needed to study the role of 11oxC19 in CD in more detail.

## Declaration of interest

M R has received honoraria for consultancies and speaker fees from Novartis, Recordati, HRA Pharma in the context of adrenostatic drugs. K S has received honoraria for consultancies from Recordati and speaker fees from Novartis. L B has received speaker fees from Novartis and Recordati. F V has received consulting fees from Recordati. K S is the PI of NeoExNET. Nicole Reisch is on the editorial board of the European Journal of Endocrinology. Nicole Reisch was not involved in the review or editorial process for this paper, on which he/she is listed as an author. The other authors report no conflicts of interest in this work.

## Funding

This work was supported by the Deutsche Forschungsgemeinschaft (Heisenberg Professorship 325768017 to N R and Projektnummer: 314061271-TRR 205 to M B, M R and N R), by a grant from the Else Kröner-Fresenius Stiftung to M R (2012_A103 and 2015_A228), by the Clinician Scientist Program RISE supported by the Eva Luise und Horst Köhler Stiftung & Else Kröner-Fresenius-Stiftung (2019_KollegSE.03 to H N and L B) and F V is supported by the Deutsche Forschungsgemeinschaft (DFG, German Research Foundation) – 413635475 – and the Munich Clinician Scientist Program (MCSP) of the LMU Munich.

## Data availability

The datasets generated during and/or analyzed during the current study are not publicly available but are available from the corresponding author on reasonable request.

## Author contribution statement

N R and K S designed the study. K S, F V, H N, L B, L T, M A, M R, C L and N R provided data. H N conducted the statistical analysis. M B, J A, J H and B K performed the laboratory analyses. H N, K S and N R drafted the manuscript. All authors provided intellectual input and read, revised and approved the final version of the manuscript. K S and N R: these authors contributed equally.
